# Stabilization through self-coupling in networks of small-world and scale-free topology

**DOI:** 10.1038/s41598-023-27809-8

**Published:** 2023-01-19

**Authors:** Jannik Luboeinski, Luis Claro, Andrés Pomi, Eduardo Mizraji

**Affiliations:** 1 Department of Computational Neuroscience, III. Institute of Physics – Biophysics, University of Göttingen, Göttingen, Germany; 2 Biophysics and Systems Biology Section, Facultad de Ciencias, Universidad de la República, Montevideo, Uruguay

**Keywords:** Dynamical systems, Network topology, Computational science, Complex networks, Phase transitions and critical phenomena

## Abstract

Mechanisms that ensure the stability of dynamical systems are of vital importance, in particular in our globalized and increasingly interconnected world. The so-called connectivity-stability dilemma denotes the theoretical finding that increased connectivity between the components of a large dynamical system drastically reduces its stability. This result has promoted controversies within ecology and other fields of biology, especially, because organisms as well as ecosystems constitute systems that are both highly connected and stable. Hence, it has been a major challenge to find ways to stabilize complex systems while preserving high connectivity at the same time. Investigating the stability of networks that exhibit small-world or scale-free topology is of particular interest, since these topologies have been found in many different types of real-world networks. Here, we use an approach to stabilize recurrent networks of small-world and scale-free topology by increasing the average self-coupling strength of the units of a network. For both topologies, we find that there is a sharp transition from instability to asymptotic stability. Then, most importantly, we find that the average self-coupling strength needed to stabilize a system increases much slower than its size. It appears that the qualitative shape of this relationship is the same for small-world and scale-free networks, while scale-free networks can require higher magnitudes of self-coupling. We further explore the stabilization of networks with Kronecker-Leskovec topology. Finally, we argue that our findings, in particular the stabilization of large recurrent networks through small increases in the unit self-regulation, are of practical importance for the stabilization of diverse types of complex systems.

## Introduction

Interacting systems with very high number of constituents and recurrent connectivity patterns are ubiquitous in both nature and technological applications. We live in a globalized world that has undergone an abrupt shortening of geographic and informational distances and an intense interdependence of economies, ecosystems, and production networks. Therefore, it has become vitally important to determine the conditions that guarantee the stability of systems with a large number of interconnected components. The stability of a dynamical system is particularly influenced by the connectivity between its constituents, which was first highlighted in the mid-twentieth century by the British psychiatrist and cyberneticist W. Ross Ashby. With theoretical investigations and empirical evidence from his “homeostat” device, he began to study how the stability of a whole dynamical system depended on the stability of its interconnected parts. One of his conclusions was that increasing the richness of connections would lower the stability^1^. Two decades later, when faster computers were available, he and Mark R. Gardner came up with a quantitative work showing that the probability of stability decreases with what they called the connectance of the system^2^. Connectance denotes the ratio of existing links with respect to the number of all possible links (if only one pair of links between each two elements is allowed). Furthermore, Gardner and Ashby discovered that larger system size would lead to a sharper transition between the stable and the unstable connectance regime.

The complex link between the connectivity and the stability of a dynamical system finally caught the attention of a broad scientific audience when Robert May presented a theoretical approach in 1972^3^. Based on Wigner’s theory of random matrices^4^, he confirmed the aforementioned conclusion of Ashby’s that stability decreases with connectance, and related it to ecology. Until then, ecologists had believed—following a paradigm established by Odum^5^, MacArthur^6^, and Elton^7^—that the increase in the number of species and the interactions between them enhanced ecosystem stability. The results of May’s work challenged the previous paradigm and opened fifty years of controversy in ecology. This so-called connectivity-stability debate still leads to a continuous production of publications on the subject (see^8,9^ for extensive reviews). Regarding ecosystems, May’s findings have been confirmed by mathematical models from the classical work of Hastings^10,11^ up to recent studies^12,13^, but at the same time others have demonstrated that real-world ecosystems exhibit reliably stable dynamics despite constant changes in connectivity^13,14^. This complex evidence has fueled the search for organizational principles and strategies that guarantee stability.

Stability is crucial not only for ecological systems but for any complex system, be it biological, social, or economic, natural or technological. The ubiquity of the connectivity-stability dilemma is reflected by examples so diverse as models of cognitive decisions in the presence of multiple uncertain choices^15^, coordination of drone swarms^16^, or the emergence of functional connectivity in the brain^17,18^. Numerous studies have been conducted in search of stabilizing factors. Ashby had already pointed out that excessive predominance of self-coupling over the interactions of the elements of the system can lead to individually stable but isolated parts^1^. Based on this, Herbert A. Simon proposed so-called near-decomposable systems which exhibit self-regulation that is strong enough to stabilize but not strong enough to cause isolation^19^. This notion relates to compartmentalization, a strategy widely occurring in biological systems from cellular to systemic levels, which serves to stabilize dynamical processes. General non-random connectivity patterns^20,21^, the variability in link strength^22^, compartmentalization^23^, the abundance of weak interactions^20,24^, and structured nonlinear connectivity^25,26^ have been investigated with respect to their capability to stabilize different dynamical systems. All of these findings notwithstanding, the underpinnings of the stability of many real-world systems remain unknown, especially of such with small-world and scale-free topology.

While Gardner and Ashby^2^ and May^3^ studied Erdős–Rényi-type random networks, real-world networks are often structured. In particular since the pioneering works of Watts and Strogatz^27^ on small-world connectivity and Barabási and Albert^28^ on scale-free degree distributions, such universal topological characteristics have been found in many complex networks. Studies considering the dynamical stability of networks of small-world and scale-free topology found no critical deviation from the behavior of (fully) random networks^29–31^, respective to the expected outcome given by the May–Wigner theorem. However, there seems to be a rather gradual transition to instability for networks in the small-world regime^29^ and a slight reduction in stability for scale-free networks^30^.

But which mechanisms lead to the stability of real-world networks? To provide an answer to this, we study here how structured networks can be stabilized efficiently by progressively increasing the self-coupling strength of their constituents. We present a systematic assessment of the stabilization of networks that have structured topologies as they are found in the real world, namely, small-world and scale-free topologies.

**Figure 1:**
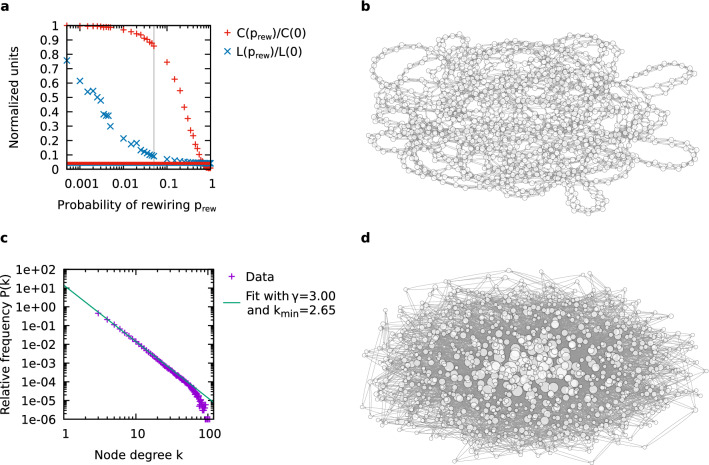
Generated small-world and scale-free networks. (**a**) Transition from regular to random topology: Watts–Strogatz clustering coefficient *C* and average shortest-path length *L* of small-world networks with $N = 1000$ and varied rewiring ratio. Both quantities are normalized with respect to the value for a regular network (i.e., $p_{{\text {rew}}} = 0$, where the Watts–Strogatz clustering coefficient is 0.500 and the average shortest path length is 125.44). The dashed red and solid blue lines indicate, for comparison, the values of *C* and *L* for the scale-free network shown in (**d**). (**b**) Sample small-world network with $N = 1000$ and $p_{{\text {rew}}} = 0.05$ (Watts–Strogatz clustering coefficient: 0.429, average shortest path length: 11.42). (**c**) Degree distribution of scale-free networks with $N = 1000$, $m_0 = 0$, and $m = 2$ (averaged over 1000 trials). The green line shows a fit function as given by Eq. (10). For fitting, all values with $k\ge 5$ were considered. See the main text for a discussion. (**d**) Sample scale-free network of $N = 1000$ (Watts–Strogatz clustering coefficient: 0.021, average shortest path length: 4.33; also cf. (**a**)). Nodes in (**b**) and (**d**) are represented by circles and node degree is indicated by circle size. Arrows represent links between nodes. Self-coupling of nodes has been masked for better visibility.

## Results

The goal of this study is to evaluate the stabilization of recurrent networks through increased self-coupling of the nodes of the network. Particularly, we have focused on networks with small-world and scale-free topology (examples are shown in Fig. 1). To this end, we used the algorithms proposed by Watts and Strogatz^27^ and Barabási and Albert^28^ to generate structured random interaction matrices exhibiting the respective topology. We then added self-coupling via the diagonal elements of these matrices. Additionally, for comparison, we have considered networks with Kronecker–Leskovec topology (see Supplementary Fig. S7). These are networks that can also exhibit small-world and scale-free properties, but are generated by a different, hierarchical algorithm (cf.^32^). In the Supplementary Information of this article, we further reproduce the results by Gardner and Ashby^2^ and thereby consider results of Erdős–Rényi-type random networks, on which much of the previous literature (see “Introduction”) has focused.

Please note that while our simulations are based on linear networks, our results also apply to nonlinear networks that can be linearized. This holds because the asymptotic stability of a linear approximation implies the asymptotic stability of corresponding nonlinear systems^33,34^. The matrices that we consider may thus be interpreted as Jacobians (first order of the Taylor expansion) governing the temporal evolution of the state vector of a dynamical system.

In this study, we refer to the asymptotic stability of dynamical systems. This stability is given if all eigenvalues of the Jacobian matrix of the system have a strictly negative real part (see “Methods”). We study how asymptotic stability behaves when network nodes are subject to self-coupling, while no nodes are removed nor added.

### Stabilizing small-world and scale-free networks by increasing self-coupling

**Figure 2:**
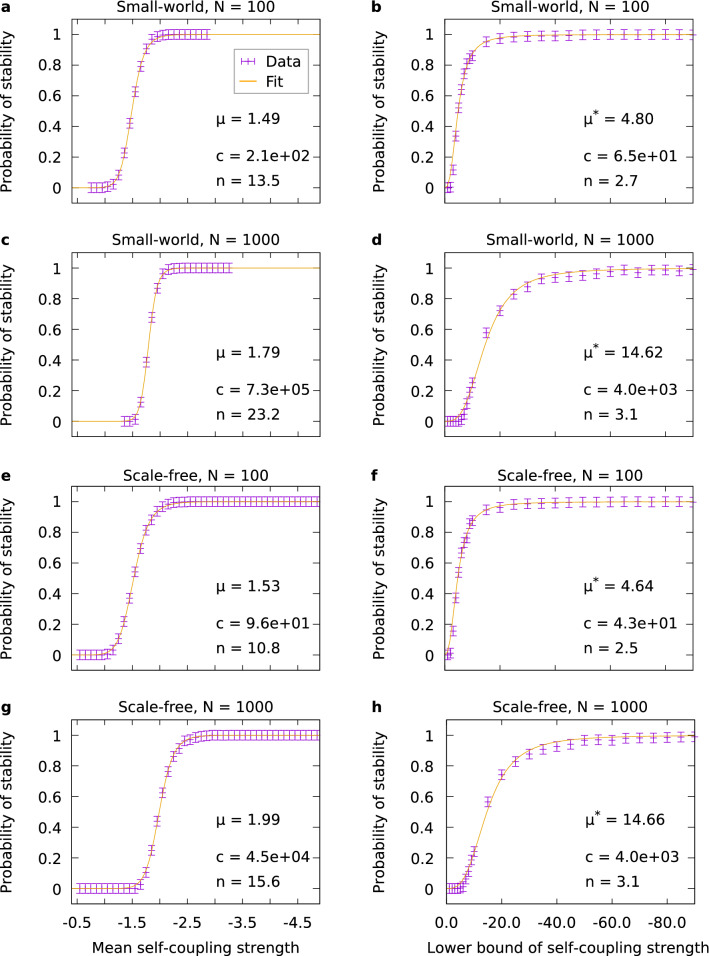
Examples of stabilization of small-world and scale-free networks. The stabilization parameter $\mu$ or $\mu ^{*}$ as well as the Hill fit parameters (cf. Eq. (1)) are given in the plots. Networks of sizes $N=100$ and $N=1000$ are shown. For each data point, the probability was averaged over 1000 trials. Error bars indicate the standard deviation estimated as described in “Methods”. (**a**,**c**,**e**,**g**) The probability of stability is plotted over the mean self-coupling strength. The self-coupling strengths were drawn from intervals of varied position but fixed size 0.9 (see main text). (**b**,**d**,**f**,**h**) The probability of stability is plotted against the lower bound of the self-coupling strength. The self-coupling strengths were drawn from an interval with lower bound varied and upper bound fixed at $-0.4$. Small-world networks in (**a**–**d**) were generated with $k_{{\text {nn}}}=4, p_{{\text {rew}}} = 5\%$, scale-free networks in (e-h) were generated with $m=2, m_0=0$ (see “Methods”).

To study how self-couplings can stabilize an otherwise unstable network of size *N*, we introduced randomly drawn self-couplings to each of the nodes of the network. Thus, we could investigate the probability of stability as a function of the mean self-coupling strength. We drew the self-coupling strength first from the interval $[-1.0,\, -0.1]$ (as in^2^). Next, we shifted this interval towards lower values while maintaining its size (the general scheme would thus be $[x-0.9,x]$; an example is $[-1.5,-0.6]$). We repeated this beyond the point where the probability of stability reached 1. We used the same procedure for small-world and scale-free networks, while we ensured that for each network size *N* the number of connections matched for both topologies (cf. “Methods”). The results for $N=100$ are shown in Fig. 2a,e and those for $N=1000$ are shown in Fig. 2c,g.

For each network size *N*, we applied a Hill function to fit the data in order to obtain the mean self-coupling strength *x* where the probability of stability $P({{\text {stable}}} \mid x)= 0.5$. The Hill function

1 $$\begin{aligned} P({{\text {stable}}} \mid x) = \frac{\left| x\right| ^{n}}{c + \left| x\right| ^{n}} \end{aligned}$$

proved to fit the data better than other possible functions like Weibull, Gompertz, Logistic function, and arc tangent.

We call the absolute value of the wanted mean self-coupling strength the *stabilization parameter* $\mu$. The stabilization parameter is given by the inverse function of the probability of stability, expressed by the fit parameters *c* and *n*:

2 $$\begin{aligned} \mu = \left| {P^{-1}({{\text {stable}}} \mid P = 0.5)}\right| = |c^{1/n}|. \end{aligned}$$

Our results show that the stabilization occurs abruptly around the inflection point, which equals the stabilization parameter (see examples for $N=100$ and $N=1000$ in Fig. 2). The sudden stabilization indicates an essentially bistable system which is very sensitive to parameter variations in between the two fixed points.

Besides the investigations with intervals of varying position, we investigated the impact of a fixed upper bound for the intervals of self-coupling strengths. To this end, we considered the upper bound fixed at $-0.4$ and only varied the lower bound (the general scheme would thus be $[x,-0.4]$), which led to an increasingly larger range of self-coupling values. We denote the stabilization parameter obtained from this different procedure $\mu ^{*}$. Examples for $N=100$ and $N=1000$ are shown in Fig. 2b,d and f,h, respectively. The results show less steep stabilization curves compared to the intervals of varying position. This indicates that an approach with larger intervals entails a slower transition toward stability.

### Stabilizing Kronecker–Leskovec networks by increasing self-coupling

To corroborate our results on small-world and scale-free topologies generated with the algorithms by Watts and Strogatz and Barabási and Albert^27,28^, we sought to compare them to results obtained from an alternative algorithm. To this end, we considered what we call Kronecker–Leskovec networks. These are self-similar networks which exhibit diameter conservation and, for large dimensions, acquire small-world and scale-free properties^32^. Furthermore, they are hierarchical by construction, which may serve to describe the hierarchical features of real-world networks (cf.^35^). Besides exhibiting such real-world characteristics, Kronecker–Leskovec graphs have the great advantage that they are easy to describe and investigate analytically.

**Figure 3:**
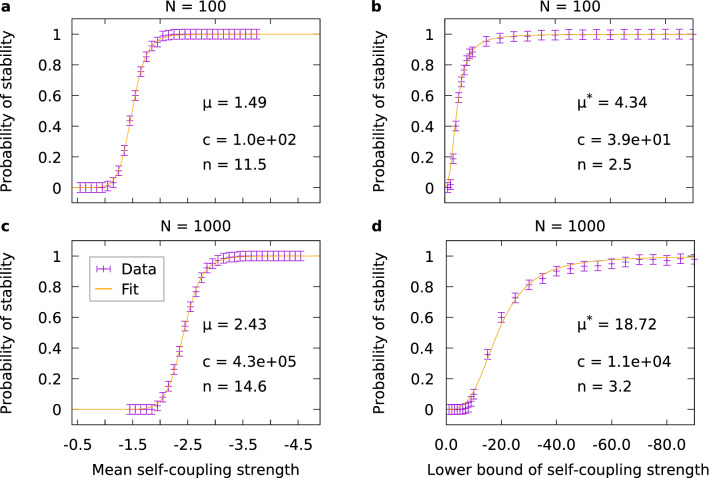
Stabilization of different Kronecker–Leskovec networks. The network size in (**a**,**b**) is $N=100$, in (**c**,**d**) $N=1000$. The self-coupling strengths for (**a**,**c**) were drawn from an interval with fixed size 0.9 and varying position, and for (**b**,**d**) from an interval with varied lower bound and upper bound fixed at $-0.4$. The stabilization parameter $\mu$ or $\mu ^{*}$ and the Hill fit parameters (cf. Eq. (1)) are provided next to the graphs. Each data point represents the average over 1000 trials. Error bars indicate the standard deviation estimated as described in “Methods”.

Following the scheme by Leskovec^32^ for the generation of real-world networks using Kronecker multiplication, we generated networks of dimensions 100 and 1000, arising from seed matrices of size 10 (cf. “Methods”). The resulting plots are shown in Fig. 3. We find that the steepness of stabilization curves for Kronecker–Leskovec networks is comparable to that for small-world and scale-free networks (cf. Fig. 2).

The nature of Kronecker–Leskovec graphs does not allow to have an arbitrary number of zeros in the final resulting matrices, which complicates the comparison between Kronecker–Leskovec networks and small-world/scale-free networks. The issue is that Kronecker–Leskovec graphs originate from a seed matrix which restricts the possible network sizes and connectivities (see “Methods”). Hence, only certain network sizes can be studied, and even at the same size *N* the number of zeros cannot be matched exactly to that of small-world and scale-free networks. The differences in the number of zeros that we want to consider here are, however, small enough to enable an approximative comparison (see Table 1). Thus, we quantitatively compare our findings for Kronecker–Leskovec networks of size 100 and 1000 to our findings for small-world and scale-free networks, which we will present in the next section.

**Table 1:** The number of zeros is slightly different in Kronecker–Leskovec networks compared to small-world/scale-free networks of the same network size *N*. For the given results, seed matrices with 82 zeros were used.

*N*	Seed matrix dim.	Kronecker power	Number of zeros in final matrix	Number of zeros in comparable small-world/scale-free networks
100	10	2	9676	9500
1000	10	3	994,168	995,000

### Stabilization depending on the network size

**Figure 4:**
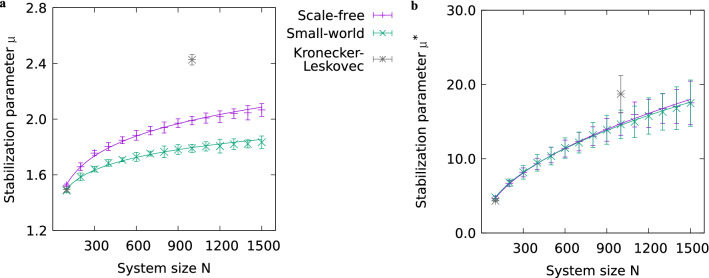
Stabilizing networks of different size and topology via self-coupling. The stabilization parameter (computed from fits to data points obtained from 1000 trials, cf. Eq. (2) and Fig. 2) is shown in relation to the network size for both small-world ($k_{{\text {nn}}}=4, p_{{\text {rew}}} = 5 \%$) and scale-free ($m=2, m_0=0$) networks. Results from Kronecker–Leskovec networks are shown for comparison. (**a**) Stabilization parameter $\mu$, referring to the mean self-coupling strength. Self-coupling strengths were drawn from small intervals of fixed size and varied position. (**b**) Stabilization parameter $\mu ^{*}$, referring to the lower bound of the self-coupling strength. Self-coupling strengths were drawn from large intervals with fixed upper bound and varied lower bound. The error bars show the error propagated from the fit parameters from which the stabilization parameter is computed. Different power-law functions fit the behavior of the stabilization parameters. See Table 2 for the fit parameters and their uncertainty.

To obtain a relationship between the stabilization parameter and the network size *N*, we performed the stability analysis that we demonstrated in Fig. 2 for a range of network sizes. The results are shown in Fig. 4. Note that here we held the generative parameters constant ($k_{{\text {nn}}}=4, p_{{\text {rew}}} = 5 \%$ for small-world, $m=2, m_0=0$ for scale-free networks), and thus, the connectance is not constant across network sizes. Essentially, we found that the stabilization parameter increases sublinearly for both small-world and scale-free networks, and for both varied (Fig. 4a) and fixed upper bound (Fig. 4b). Furthermore, for varied upper bound, we found quantitative differences between small-world and scale-free topology.

For the considered range of network sizes from $N = 100$ to $N = 1500$, the power-law function

3 $$\begin{aligned} \mu (N) = A \cdot \left( N - 1\right) ^p \end{aligned}$$

properly fits the course of the stabilization parameter, which is sublinear with $A>0$ and $p<1$, as shown in Table 2. At the same time, the function does account for the constraint that for $N = 1$, stabilization ought to happen at self-coupling strength 0. As indicated by the error values in Table 2, the fits that we obtained are very precise for both small-world networks and scale-free networks. Although some data points of the scale-free networks in Fig. 4a exhibit small visible deviations from the fit function, these remain well within the error range of the data points. Compared to our scale-free networks generated with the preferential attachment algorithm with parameters $m=2$ and $m_0=0$ (see Fig. 4), we obtained slightly different results for approximate scale-free networks generated with alternative parameters $m=4$ and $m_0=N/2$. The corresponding plots are shown in Supplementary Fig. S6.

From the results given in Fig. 4 and in Table 2, we can further evaluate the impact of the spread of the self-coupling strength values as defined by small intervals (varied upper bound/position) versus large intervals (fixed upper bound). For large intervals, we find that small-world and scale-free networks become stabilized at almost the same values of the stabilization parameter (absolute values of self-coupling). For small intervals, however, small-world networks are stabilized with significantly lower values as compared to scale-free networks. The networks generated with the preferential attachment algorithm with alternative parameter values yet yield other results for small intervals (cf. Supplementary Fig. S6a and Table 2)—they behave more like our small-world networks, which may be explained by the fact that for these alternative networks the scale-free property is not as pronounced as for our standard scale-free networks (compare Supplementary Fig. S4a with Fig. 1c).

As pointed out in the previous subsection, we could not investigate the same number of data points for Kronecker–Leskovec networks as for small-world and scale-free networks. Nevertheless, for comparison, we provide results for Kronecker–Leskovec networks of two different sizes in Fig. 4. We see that at $N=100$, there is no substantial difference to the standard small-world and scale-free networks, but at $N=1000$, the Kronecker–Leskovec networks require much stronger stabilization than the standard small-world and scale-free networks. This may be due to the fact that the Kronecker–Leskovec networks exhibit a particularly high amount of hub nodes and low average shortest-path length. However, they do not feature scale-free characteristics entirely (see Supplementary Fig. S7a).

**Table 2:** Resulting parameter values for power-law fit of stabilization parameter over network size (cf. Eq. (3)). The upper subtable relates to the stabilization parameter $\mu$, which means that the self-coupling strength was drawn from intervals with fixed size and varied position. The lower subtable relates to the stabilization parameter $\mu ^{*}$, which means that the self-coupling strength was drawn from intervals with fixed upper bound. Uncertainties are given by the asymptotic standard error (computed via gnuplot, cf. “Methods”). Corresponding plots are shown in Fig. 4a, Supplementary Fig. S6a (upper table) and in Fig. 4b, Supplementary Fig. S6b (lower table).

Interval type	Topology	*A*	*p*
Varied position	Small-world	$1.045 \pm 0.013$	$0.078 \pm 0.002$
	Scale-free ($m=2, m_0=0$)	$0.906 \pm 0.008$	$0.114 \pm 0.002$
	Approximate scale-free ($m=4, m_0=N/2$)	$1.098 \pm 0.019$	$0.080 \pm 0.003$

Interval type	Topology	$A^{*}$	$p^{*}$
Fixed upper bound	Small-world	$0.537 \pm 0.006$	$0.477 \pm 0.002$
	Scale-free ($m=2, m_0=0$)	$0.481 \pm 0.011$	$0.495 \pm 0.004$
	Approximate scale-free ($m=4, m_0=N/2$)	$0.559 \pm 0.007$	$0.474 \pm 0.002$

To summarize, we found a power-law dependence between the stabilization parameter and the size of small-world and scale-free networks (within a certain range of sizes). While both topologies exhibit a power-law characteristic, there are quantitative differences between the small-world and scale-free topology for small intervals (with varied upper bound/position). By capturing the quantitative properties of our simulation results (see Table 2), we provide empirical rules that can be used to predict the stability of a system given its topology and size. Nevertheless, further studies may investigate if deviations from the power-law description will occur at larger network sizes (see “Discussion”).

### Application to real-world systems

There is a wide variety of natural, social, technological, as well as formal systems to which we expect our results to apply. Note that although most dynamical systems in the real world are nonlinear, our generic results nonetheless apply to any nonlinear system that can be linearized. In this hypothesis subsection, we will consider three examples to demonstrate how our general results may be applied. The examples are summarized in Table 3.

**Table 3:** Analogies between abstract networks and real-world applications. Three examples from different fields (neuroscience, electrical engineering, and epidemiology) are given.

Abstract property	Networks in the brain	Smart power grids	Infection dynamics
Nodes	Neurons; populations of neurons	Power plants and other participants, including households	Human individuals, possibly animals and inanimate entities
Weights	Effective excitation/inhibition	Effective demand/supply of electrical power	Effective contribution to the infection dynamics by physical interaction
Negative self-coupling	Effective self-inhibition/-excitation	Effective self-supply/-consumption	Vaccination; immune defense
Instability can lead to…	Epileptic seizure (for excessive excitation); suppressed activity (for excessive inhibition)	Power outage; waste of energy	Uncontrolled wave of infections

We will start by considering the application to biological neural networks. In studies using functional magnetic resonance imaging (fMRI) or magnetoencephalography/electroencephalography (MEG/EEG), the functional network connectivity of the human brain has been found to exhibit small-world features. At the same time, the anatomical structure of neural connectivity has also been found to follow a small-world topology^17,36–38^. Besides that, truncated scale-free degree distributions have been found in functional connectivity^36,37^. Despite these findings, much remains to be learned about the dependence of brain function on small-world and scale-free topologies, particularly with regard to the understanding of neuropsychiatric diseases or drug effects. An important aspect is that a certain degree of stability is necessary for the functionality of neural dynamics, which is disrupted, for example, in epilepsy^39^. Regarding this, the results presented here can serve to assess the stabilization properties of a network of, for example, *N* neurons or *N* interacting brain areas.

To assess the stability of a system of *N* neurons or neuronal populations, we can describe their approximated firing rate activities by the vector *r* (cf.^40^):

4 $$\begin{aligned} \tau \frac{dr}{dt} = w \cdot r. \end{aligned}$$

Here, the timescale of the dynamics is adjusted by the constant $\tau$. The couplings between the populations are described by the $N \times N$ weight matrix *w*. If now the matrix exhibits suitable small-world or scale-free characteristics, and the weights are drawn from the interval $[-1.0,+1.0]$, and *N* does not exceed 1500, the stabilization properties of the approximated network dynamics can be assessed precisely by the empirical rules that we have presented in the previous section (Table 2).

To consider neural systems beyond this, functional or effective connectivity data (cf.^38,41,42^) may be assessed with our stability predictions in future studies. Furthermore, our results may be compared to studies targeting the storage capacity of attractor neural networks (cf.^43,44^) or to studies directly targeting the interactions between memory representations (cf.^45–47^).

Next, we want to see how our results can relate to the dynamics of smart power grids. Note that in these, participants can be both producer and consumer at the same time. Thus, all participants can in principle produce energy for their own supply and beyond. In traditional power grids, however, only a few large power plants produce energy for a large number of consumers, and thus, only the power plants exhibit self-supply (self-coupling). The stability of power grids can be affected greatly by tuning the self-coupling of the nodes/participants (cf.^48–50^). For instance, a power plant that does not block electrical energy from entering it from outside will likely produce a power outage. Similarly, a small consumer not restricting their received energy can also cause instabilities, even though these would probably have less severe effects for the whole system in a realistic grid.

Since our results are based on self-coupling for all nodes, they cannot be applied to traditional power grids. However, we can consider the impact of largely decentralized, small-world, smart grids versus relatively centralized, scale-free, smart grids. Our results predict that the first should be stabilized more easily, whereas the latter should be more prone to power outages. This is supported by previous studies showing that decentralized grids are more likely to be stable while centralized grids are indeed more prone to power outages^48,51^.

It may also be interesting to note that a different application of our results might relate to the issue of privacy in smart grids, where stability may be investigated in the sense of the identification of a person participating in the smart grid (cf.^52^).

Lastly, we would like to consider an example of infection dynamics. Many possible instabilities with respect to these have recently become empirically evident throughout the Covid-19 pandemic. To stabilize the infection dynamics of this disease, face masks^53^, travel restrictions^54^, and vaccinations^55,56^ have proven to be useful means.

In the framework that we have studied here, the effect of such means may be approximated as follows: 1. negative self-coupling strength may correspond to the degree of protection achieved by vaccination; 2. positive coupling from one node to another may represent significantly enhanced risk of infection; 3. negative coupling from one node to another may represent lowered risk due to the presence of protection, which contributes to the decay of a wave of infections. Thus, the coupling between nodes (persons) can be thought to depend on the wearing of a face mask, the physical distance between persons, and the vaccination status.

In conclusion, our findings on the stabilization dynamics of small-world and scale-free networks indicate that infection-preventing measures may have a different impact depending on the topology of the social network that is considered. In particular, our results make the prediction that, to control the spread of infections in scale-free-type networks like the air traffic network (cf.^57^), stronger measures will be required as compared to small-world networks like the neighborhood structure of a city or the highway traffic network (cf.^58^).

Taken together, our results can serve as predictions for various dynamical systems across fields. It has, nevertheless, to be shown in specialized studies how well the prediction matches a particular real-world scenario.

## Discussion

We have carried out a systematic study of the stabilization of recurrently connected networks through self-coupling of the network constituents. We investigated this stabilization with respect to small-world and scale-free network topology. In addition, we considered the stabilization of Kronecker–Leskovec graphs of comparable size, and we presented hypothetical applications of our theoretical findings.

It is important to acknowledge that graphs may exhibit small-world and scale-free characteristics at the same time. Here, we have chosen the approach of separating the two topologies in order to demonstrate their individual impact. To ensure that the networks that we considered exclusively feature small-world or scale-free characteristics, we measured the clustering coefficient and verified that it was significantly higher for small-world than for scale-free networks (cf. Fig. 1a). On the other hand, we also verified that the heavy-tailed degree distribution that is typical for scale-free networks (cf. Fig. 1c) did not occur in the small-world networks (cf. Supplementary Fig. S3). We should also note here that the degree distribution of scale-free networks offers an interesting opportunity for future studies, as it exhibits highly-connected nodes, so-called hubs, which are opposed to a majority of nodes with very low connectivity. In particular, it would be interesting to explore whether selective reinforcement of the self-coupling of hubs could serve to stabilize entire networks.

In the following, we shall highlight three key findings of our study. First, we found the existence of an abrupt transition to stability with increasing average absolute value of the elements of the main diagonal of the interaction matrices. This threshold-like behavior is relatively independent of the network structure. Considering the asymptotic stability over the mean self-coupling strength (Figs. 2 and 3) shows that for weak self-coupling no stability is possible, while after a short threshold region a regime follows in which all trials are stable. In that regime, the network dynamics seem to be dominated by self-coupling, thus being stabilized as proposed by Ashby in his original paper in 1950^1^. Note that self-coupling can be interpreted as a modification of the effective timescale of the respective node, where stronger negative self-coupling leads to faster inhibitory dynamics and thereby to stability. Due to the numerical character of our study, we had to decide for specific intervals to draw the self-coupling strength from. We took our choice so as to cover two relatively distinct cases (relatively small and large intervals). As our results show, these two kinds of intervals indeed yield distinct behavior. We further found that the threshold region is modeled best by a sigmoidal Hill function, although lacking some accuracy in the part where stability begins to rise. For some simulations with self-coupling strength drawn from an interval of fixed size, fitting with a Logistic function could improve accuracy, however, the Hill function was also used here for consistency.

Second, the stabilization parameter increases sublinearly as a function of the network size. We showed that in the considered range of network sizes ($N = 100$ to $N = 1500$), the function can be described by a power law. This result is of particular interest because it demonstrates that the stabilization of larger systems can be achieved relatively more easily than the stabilization of smaller systems. It might be possible that (deviating from a power-law description) the stabilization parameter approaches a limit for large *N*, which would mean that there were one threshold for very large systems that would certainly stabilize the system. Nevertheless, such a particular limit seems implausible. The empirical power-law rules that we found for a range of network sizes might hold, in contrast, as a general law. To solidify this hypothesis, the collection of more data will be necessary. Analytical investigations may, in addition, serve to uncover a general law and to yield further interesting insights into the asymptotic stability behavior of complex systems (also cf.^25^). Existing analytical results already hint at a possible power-law stabilization function for linearized systems (cf.^59^). Furthermore, the May–Wigner theorem^3,10,11,60^ may serve to approach the relationship between stabilization and network size, although there are several restrictions (cf.^13,61^).

Third, the topology of the network influences the magnitude of self-coupling required to stabilize the system. We found that scale-free networks need stronger self-coupling for stabilization than small-world networks. A major difference between these two topologies is that while the average shortest-path length is low for both small-world and scale-free networks, the clustering coefficient of small-world networks is a lot higher than that of scale-free networks (cf. Fig. 1). Hence, high clustering may contribute to stabilization (also cf. Supplementary Fig. S2). Although there is a substantial difference in magnitude, it is interesting that small-world and scale-free networks show very much the same *qualitative* behavior with respect to stabilization as a function of the network size (Fig. 4). For comparison, we also considered the stabilization of Kronecker–Leskovec graphs at two different network sizes. From the given samples, it seems that the Kronecker–Leskovec topology may require even higher self-coupling to acquire stability as compared to small-world and scale-free topologies. Besides that, Kronecker–Leskovec networks can easily be described in an analytical manner (cf. “Methods”), and thus, the given results are eligible to be compared with analytical approaches in the future.

As a general conclusion, the stabilization parameter for different network topologies depends on the network size in a sublinear manner, which reveals an intriguing path to stability: The stabilization of large recurrent networks can be achieved with small increases in the self-regulation of the elements of the system because these yield a supralinear increase in the resilience of the whole system. This is a finding that should not be underestimated. As a result of technological advances, we live in a hyper-connected world which exhibits increasing risks of ecological, technological, and social instability at a global level. We have most recently experienced, through the Covid-19 pandemic, that decreased mobility (i.e., isolation through decreased connectivity) enables to control the spread of infections. At the same time, it could also be controlled by self-inhibition of the infection dynamics, for example, through the use of face masks and vaccinations (cf.^53,56^).

The general idea that component self-regulation (i.e., the intrinsic stability of isolated system constituents) is important for the stability of a whole dynamical system has been around at least since the seminal work by Ashby in 1950^1^. In the present work, we have studied the effect of reinforcing self-coupling on the elements of otherwise unstable complex systems. We have considered systems with structured topologies as they are found in very different areas of the real world, and we obtained results that suggest the importance of this path to stabilization. Thus, the predictions emerging from our work—mainly, that small increases in the local stability of the components can have a significant effect on the global stability of the system—should be important to researchers from various disciplines and deserve further analysis.

## Methods

### Determining the asymptotic system stability

The asymptotic stability of a dynamical system is evaluated considering the eigenvalues of the interaction matrix that represents the first-order (i.e., linear) approximation of the system. If the real part of any of the eigenvalues is positive, the system is unstable.

The eigenvalues of the system can, in general, be determined in different ways. A first approach is to compute the characteristic polynomial of the interaction matrix and to solve for its zeros using Horner’s/Newton’s method. A second approach is to use the characteristic polynomial to apply the Routh–Hurwitz criterion as it was done by Gardner and Ashby^2^. Both of these approaches take very long because computing the characteristic polynomial is very expensive. A third approach, which we take here, is to compute the eigenvalues directly from the interaction matrix employing the Francis’ QR double-shift algorithm (as implemented in the GNU Scientific Library). Nevertheless, to validate our implementation, we compared the results of all three methods and found them to be equal.

We compute the *probability of stability* by dividing the number of trials for which the system was asymptotically stable by the total number of trials. Given that the system is either stable or unstable, we can denote these cases by 1 and 0, respectively, and the outcome of a trial *i* is given by

5 $$\begin{aligned} s_i \in \{0,1\}. \end{aligned}$$

The probability of stability is then

6 $$\begin{aligned} P({{\text {stable}}}) := \langle s \rangle = \frac{1}{T} \sum \limits _{i=1}^{T} s_i, \end{aligned}$$

where *T* is the total number of trials. The uncertainty of this measure is computed as follows. The standard deviation of the outcome of one trial is given by

7 $$\begin{aligned} \Delta s_i := \sqrt{\langle s^2 \rangle - \langle s \rangle ^2} = \sqrt{\langle s \rangle - \langle s \rangle ^2} = \sqrt{P({{\text {stable}}})\cdot (1-P({{\text {stable}}}))}, \end{aligned}$$

where we have used Eqs. (5) and (6). The standard deviation of the probability of stability can then be approximated by means of Gaussian error propagation:

8 $$\begin{aligned} \Delta P({{\text {stable}}}) := \sqrt{\sum \limits _{i=1}^{T}\left( \Delta s_i\,\frac{\partial p}{\partial s_i}\right) ^2} = \sqrt{\sum \limits _{i=1}^{T}\left( \frac{\Delta s_i}{T}\right) ^2} = \sqrt{T\cdot \left( \frac{\Delta s_i}{T}\right) ^2} = \frac{\Delta s_i}{\sqrt{T}}\le \frac{1}{\sqrt{T}}, \end{aligned}$$

where we have used Eqs. (6) and (7). We safely estimate this measure here by using its upper bound $1/\sqrt{T}$.

### Generation of small-world topology

We generate small-world networks in a manner that is similar to the scheme presented by Watts and Strogatz in 1998^27^. To this end, we initially create a regular network of *N* nodes, with each node having connections to its $k_{{\text {nn}}}$ nearest neighbors. Then, an exact number of rewirings is performed. Although Watts and Strogatz used, in contrast, an average number of rewirings, the rules for rewiring are the same: no edge may be rewired twice, and there may only be one edge for each pair of nodes. The procedure leads to undirected graphs. To test the small-world property of the generated networks, we have plotted the averaged clustering coefficient and the average shortest-path length against the number of rewirings (see Fig. 1a). Finally, we turn the simple, undirected graphs obtained from the procedure described above into multigraphs with bidirectional (antiparallel) links. An example graph of such a small-world network is shown in Fig. 1b. We draw the weight of the antiparallel links—individually for each direction—from a uniform random distribution in the interval between $-1.0$ and $+1.0$. This yields bidirectional but asymmetric graphs, which can relate to a vast variety of real-world systems. Note that the corresponding matrices are structurally symmetric (with respect to their values being non-zero or zero). Finally, we introduce self-coupling by adding a loop of negative weight to each node (more details are given in the “Results” section).

### Generation of scale-free topology

We produce scale-free networks by means of the ‘preferential attachment’ algorithm described by Barabási and Albert^28^. This algorithm is based on the finding that in growing scale-free networks, highly-connected nodes receive more new connections than sparsely connected ones. To obtain a network of *N* nodes, we start from a population of $m_0$ unconnected nodes and add $N - m_0$ nodes, each one connecting with *m* edges to already existing nodes. In doing so, the probability of connecting to a specific node *i* is proportional to its degree $k_i$:

9 $$\begin{aligned} \Pi \left( k_i\right) = \frac{k_i + 1}{\sum _{j} \left( k_j + 1\right) }. \end{aligned}$$

The addition of unity in the above equation is necessary to avoid an initial attachment probability of zero. To obtain bidirectional but asymmetric connectivity, as for our small-world networks (see above), link strengths are individually drawn for each direction from a uniform random distribution in the interval between $-1.0$ and $+1.0$. We finally introduce self-coupling by adding a loop of negative weight to each node (see the “Results” section for more details).

We verified the scale-free property by fitting the following power-law function to the averaged-over-trials degree distribution of all nodes^57^:

10 $$\begin{aligned} P(k) = \left( \gamma -1\right) \cdot k_{{{\text {min}}}}^{\gamma -1} \cdot k^{-\gamma }. \end{aligned}$$

In this equation, $k_{{\text {min}}}$ represents the degree where the presumed maximum of the distribution occurs, while $\gamma$ is the slope parameter. Scale-free networks typically exhibit $2 \lesssim \gamma \lesssim 3$^57^, which we have found is the case for our networks (cf. Fig. 1c). An example graph of a generated scale-world network is shown in Fig. 1d.

### Generation of Kronecker–Leskovec topology

To generate Kronecker–Leskovec networks, we follow the scheme described by Leskovec^32^ which is based on Kronecker multiplication of seed matrices. For each trial, we start from a randomly generated symmetric seed matrix *S* of dimension 10. This enables us to consider networks of dimensions 100 and 1000. We obtain a Kronecker–Leskovec matrix $K_2$ of dimension 100 by the following Kronecker product:

11 $$\begin{aligned} K_2 = S \otimes S. \end{aligned}$$

To obtain a Kronecker–Leskovec matrix $K_3$ of dimension 1000, we perform the Kronecker product once more:

12 $$\begin{aligned} K_3 = K_2 \otimes S. \end{aligned}$$

We introduce self-coupling by adding a loop of negative weight to each node (see the “Results” section for more details).

The degree distribution of the Kronecker–Leskovec networks turns out to have certain similarity with that of scale-free networks, which is shown in Supplementary Fig. S7, along with an example graph of a generated Kronecker–Leskovec network.

### Parameters for similar connectance across topologies

To be able to compare the results of small-world networks and scale-free networks, the same connectance has to be used. For small-world networks, the connectance is simply given by:

13 $$\begin{aligned} C_{{{\text {sw}}}} = \frac{ k_{{\text {nn}}} \cdot N }{ N^2 -N } = \frac{ k_{{\text {nn}}} }{ N-1 }. \end{aligned}$$

For scale-free networks, on the other hand, the connectance is given by:

14 $$\begin{aligned} C_{{{\text {sf}}}} = \frac{ 2m \cdot \left( N - m_0 \right) }{ N^2 -N }. \end{aligned}$$

We find the parameter values that we need to achieve the same connectance for both topologies by solving the equation

15 $$\begin{aligned} C_{{{\text {sw}}}} = C_{{{\text {sf}}}}. \end{aligned}$$

Inserting Eqs. (13) and (14) we obtain:

16 $$\begin{aligned} \frac{k_{{\text {nn}}}}{N-1} = \frac{2m\,(N-m_0)}{N^2-N}, \end{aligned}$$

which we can rearrange to obtain:

17 $$\begin{aligned} 0 = (2 m - k_{{\text {nn}}})\,N - 2 m_0 \, m. \end{aligned}$$

One solution of this equation is given by $k_{{\text {nn}}}=2m$ and $m_0=0$. Based on this, we chose to use the parameter values $k_{{\text {nn}}}=4$, $m=2$, and $m_0=0$ primarily (also see Supplementary Fig. S2 on the impact of $k_{{\text {nn}}}$ and rewiring for small-world networks). For these values, the preferential attachment algorithm often leads to a few ($<10$) edges less than expected. This may be due to the small number of *m* and the value 0 for $m_0$. We obtained, nevertheless, typical scale-free degree distributions with only slight deviations at high degrees. An example plot is shown in Fig. 1c.

Another solution for Eq. (17), yielding equal connectance values $C_{{{\text {sw}}}} = C_{{{\text {sf}}}}$, is given by $k_{{\text {nn}}} = m$ and $m_0 = N/2$. Therefore, we also considered the parameter setting $m = k_{{\text {nn}}} = 4$ to generate networks with the preferential attachment algorithm, which yields only approximate scale-free characteristics (see Supplementary Figs. S4, S5 and S6). This setting features a high number of initially unconnected nodes, e.g., $m_0=500$ for $N=1000$. While the degree distribution for this setting deviates from the scale-free characteristic at low and high degrees, the overall slope of the distribution is in the typical range of scale-free networks (cf. Supplementary Fig. S4a). Such deviations are not unusual for networks exhibiting scale-free properties (cf.^57^) and could be caused by the high number of initially unconnected nodes. Nevertheless, both types of networks generated with the preferential attachment algorithm exhibited clustering coefficients and average shortest-path lengths in the expected regime (also cf. Fig. 1a and Supplementary Fig. S4a).

For Kronecker–Leskovec matrices to have a number of zeros comparable to our small-world and scale-free matrices, we fixed the number of zeros in the seed matrix *S* at 82, which results in the values given in Table 1.

### Computational implementation and software used

We used C++ in the ISO 2011 standard to implement our simulations. Networks were represented by means of a matrix class. All nodes were given a self-coupling of random strength, drawn from a uniform distribution over a specific interval (see above). Random numbers were generated using the generator minstd_rand0 of the C++ standard library, while the system time served as the seed. We implemented a loop in our code which ensured that for each distribution a unique seed was used. Unless stated otherwise, we ran each simulation 1000 times to obtain mean and error estimates of the respective quantities. To compile and link the code, we employed g++ in version 7.4.0 with boost in version 1.65.1. For the computation of eigenvalues, we employed GNU Scientific Library (GSL) 2.5. Our code is freely available under the terms of the GNU General Public License v3.0^62^.

For the creation of plots we used gnuplot 5.0.4, only for graphs we used Pajek64 in version 5.14 with the Kamada–Kawai Free algorithm. Pajek64 also served to compute average shortest-path lengths and Watts–Strogatz clustering coefficients. For fitting, we employed gnuplot as well.

## Supplementary Information

Supplementary Information.

## Data Availability

We have made our program code freely available^62^. The code enables to reproduce all results presented in this study. Furthermore, the datasets used and analyzed for the current study are available from the corresponding author upon request.
