# Supplementary material for: Stabilization through self-coupling in networks of small-world and scale-free topology
Source: Sci Rep. 2023 Jan 19;13:1089. doi: 10.1038/s41598-023-27809-8 (PMC9851597; doi:10.1038/s41598-023-27809-8)
Supplement: Supplementary file 1 — Supplementary Information. [file 41598_2023_27809_MOESM1_ESM.pdf]

Supplementary Information: Stabilization through self-coupling in networks of small-world and scale-free topology

Jannik Luboeinski[1,2], Luis Claro[2], Andrés Pomi[2], Eduardo Mizraji[2]

[1]Department of Computational Neuroscience, III. Institute of Physics – Biophysics, University of Göttingen, Göttingen, Germany

[2]Biophysics and Systems Biology Section, Facultad de Ciencias, Universidad de la República, Montevideo, Uruguay

*Correspondence: mail@jlubo.net

2022-12-24

Reproduction of results on Erdős-Rényi-type random networks

As a first step to validate our implementation we sought to reproduce the results of Gardner and Ashby (1970). Therefore, we generated graphs with randomly chosen links. Two different algorithms were used for this occasion. The first algorithm distributes links in a sample network such that on average (over samples/trials) a certain connectance value is reached. This is the method used by Gardner and Ashby, corresponding to Erdős-Rényi random graphs. The second algorithm distributes an exact number of links in every sample network, thus leading to exactly the same connectance for every trial. This corresponds to Erdős-Rényi-Gilbert random graphs (cf. Fienberg, 2012). Both algorithms lead to directed graphs whose link strengths are drawn from a uniform random distribution in the interval between -1.0 and $+1.0$. Self-coupling strengths are drawn from in between -1.0 and -0.1 (also cf. Methods in the main article). It may be noteworthy that May (1972) used normally-distributed random numbers in his theoretical study, albeit referring to the results that Gardner and Ashby had obtained with uniformly-distributed coupling strengths.

The resulting graphs for an average as well as for an exact number of connections are shown in Fig. S1a,b. Considering the general trend, there is no difference between these graphs. There is, however, a difference between the results presented here and those presented by Gardner and Ashby (1970). Their curves for $N = 7$ and $N = 10$ drop quite sharply to zero at 40 % and 13 %, respectively, while our results suggest a slower descent. Since we could cross-validate the simulation results obtained from our C++ implementation with a second implementation in Matlab, it seems that the results obtained by Gardner and Ashby might have been biased by the small number of trials that they had been able to use back in 1970.

Another important point is to compare the freely directed graphs considered in Fig. S1a,b and the bidirectional asymmetric graphs considered in Fig. S1c,d. There is a certain deviation at lower connectance values, but the curves converge to the same stability value at connectance 100 %, as one would expect. While the freely directed graphs exhibit slightly increased stability, this is negligible for the purpose of our study.

Please note that for all results in the present study, except those shown in Fig. S1a,c, we used "exact number" (Erdős-Rényi-Gilbert-type) algorithms. Moreover, except for the freely directed graphs used for Fig. S1a,b, we considered bidirectional asymmetric graphs.

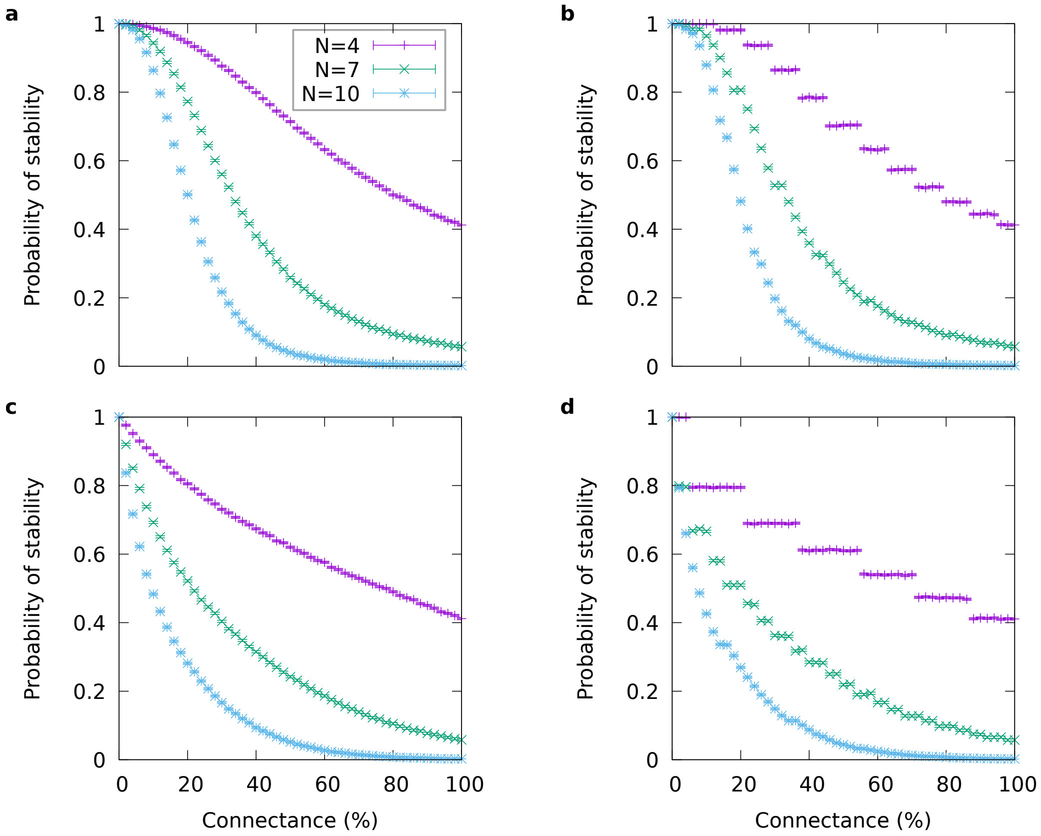

Supplementary Figure S1: **Reproduction of the results of Gardner and Ashby (1970) using $\mathbf{10^5}$ trials. (a,b)** As in the original study (Gardner and Ashby, 1970), freely directed graphs are used. **(c,d)** Bidirectional asymmetric graphs are used, meaning that for every link there is an antiparallel link with a different coupling strength. In (a,c) each connectance value leads to a certain number of connections, on average (Erdős-Rényi-type graphs). In (b,d), each connectance value corresponds to an exact number of connections (Erdős-Rényi-Gilbert-type graphs; cf. Fienberg, 2012). This elicits discretized 'blocks' of values. Mostly too small to be visible, error bars indicate the estimated standard deviation (described in Methods).

Variation of connectance and rewiring number for small-world networks

To explore the stability of small-world networks depending on the connection parameters used to construct the networks, we determined the probability of stability of small-world networks with varied rewiring number R and varied number of nearest-neighbor connections k_{nn}. This enabled us to examine the probability of stability as a function of the connectance C_{sw}, which is given by (cf. Eq. 9 in the main article):

$$C_{\mathrm{sw}} = \frac{k_{\mathrm{nn}}}{N-1} \cdot 100\%.$$

Sample graphs are shown in Fig. S2 for different rewiring numbers. The number of data points is restricted by the fact that k_{nn} can only take even integer values. Although the curves shown in Fig. S2 represent regular (for $R = 0$) or small-world networks (for $R > 0$), they exhibit the same principal behavior as random networks – the probability of stability declines with increasing connectance. Nevertheless, differences occur across different numbers of rewirings. At low connectance values, all networks are stable. At moderate connectance values, rewiring slightly increases the stability. Eventually, at high connectance values, all networks become unstable.

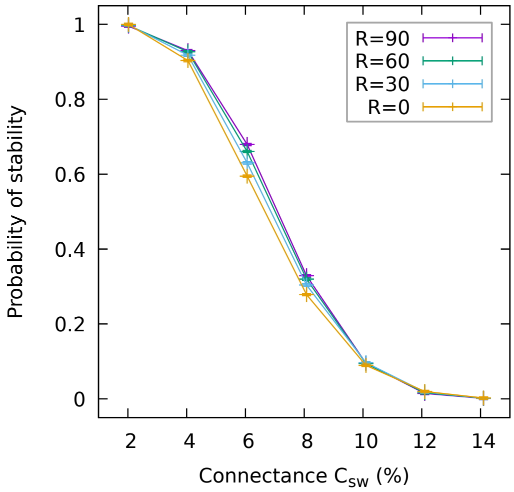

Supplementary Figure S2: **Impact of connectance and rewiring number on the probability of stability of small-world networks.** The probability of stability is plotted against the connectance (here, $\approx k_{\mathrm{nn}}$) for networks of $N = 100$ nodes. Different curves show data for different numbers R of rewired connections. For each value, 10^5 trials were used. Error bars indicate the standard deviation (mostly too small to be visible) estimated as described in Methods. Self-coupling strengths were drawn from a uniform distribution between -2.2 and -1.3. The construction of small-world networks is described in the Methods section of the main article.

To verify that the considered small-world networks did not exhibit a substantial number of high-degree nodes (which would constitute additional scale-free characteristics – see discussion in the main article), we had to check that the degree distribution of the small-world networks would be sufficiently narrow. The plot in Fig. S3a shows that if there is no rewiring, all nodes have a degree of 5 (4 nearest-neighbor connections plus the self-coupling). With increasing rewiring

number R, the distribution broadens and the node degree ranges between 1 and 12 (Fig. S3b-e), but there is still no substantial number of high-degree nodes (also cf. Fig. 1c in the main article).

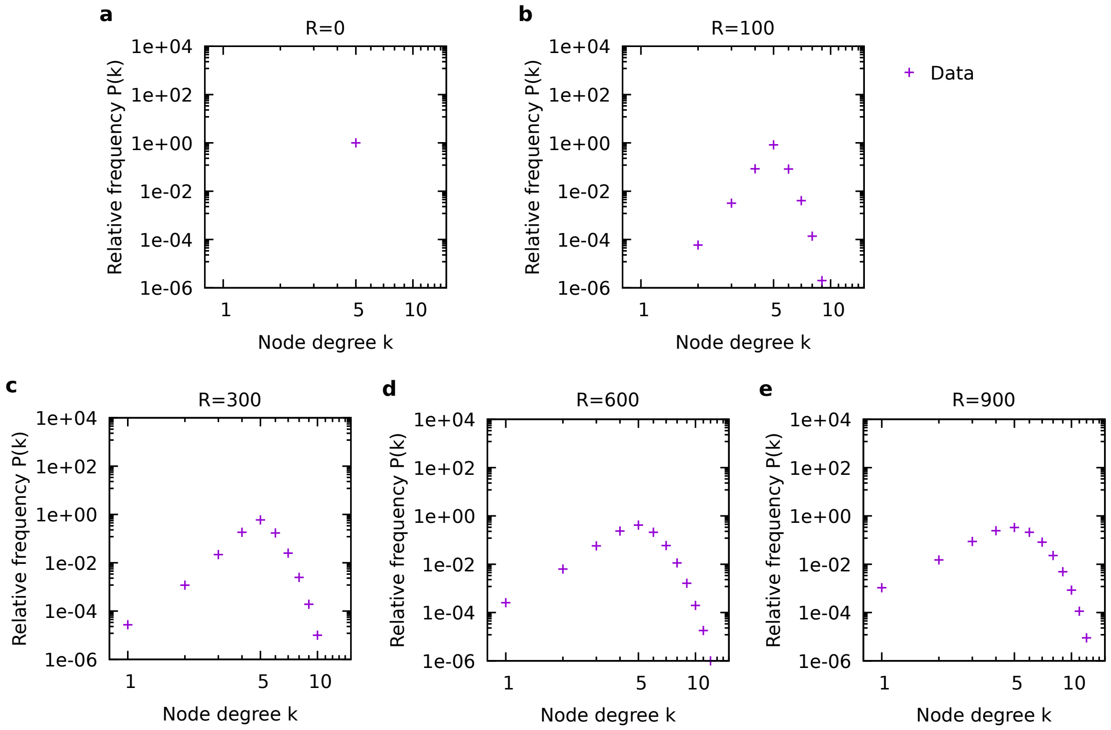

Supplementary Figure S3: **Degree distribution of small-world networks with varied rewiring number.** Averaged over 1000 networks with size $N = 1000$ and number of nearest-neighbor nodes $k_{\mathrm{nn}} = 4$. Number R of rewired connections: **(a)** 0 (equals $p_{\mathrm{rew}} = 0$); **(b)** 100 (equals $p_{\mathrm{rew}} = 0.05$); **(c)** 300 (equals $p_{\mathrm{rew}} = 0.15$); **(d)** 600 (equals $p_{\mathrm{rew}} = 0.30$); **(e)** 900 (equals $p_{\mathrm{rew}} = 0.45$).

Alternative parameters for the preferential attachment algorithm

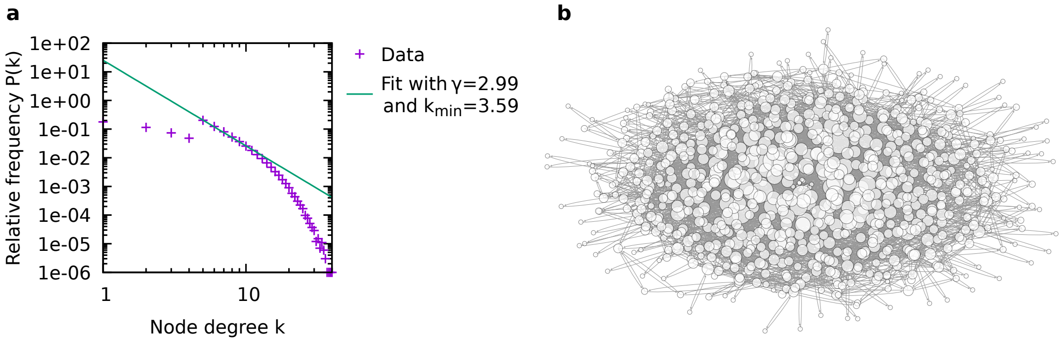

Supplementary Figure S4: **Networks generated with alternative preferential attachment parameters.** **(a)** Degree distribution of networks with $N = 1000$, $m_0 = 500$, and $m = 4$ (averaged over 1000 trials). The green line shows a fit function as given by Eq. 10 in the main article. For fitting, all values with $k \geq 5$ were considered. See the text in the main article for discussion. **(b)** Sample network of $N = 1000$ (Watts-Strogatz clustering coefficient: 0.004, average shortest path length: 4.25; also cf. Fig. 1a in the main article). Nodes are represented by circles and node degree is indicated by circle size. Arrows represent links between different nodes. Self-coupling of nodes has been masked for better visibility.

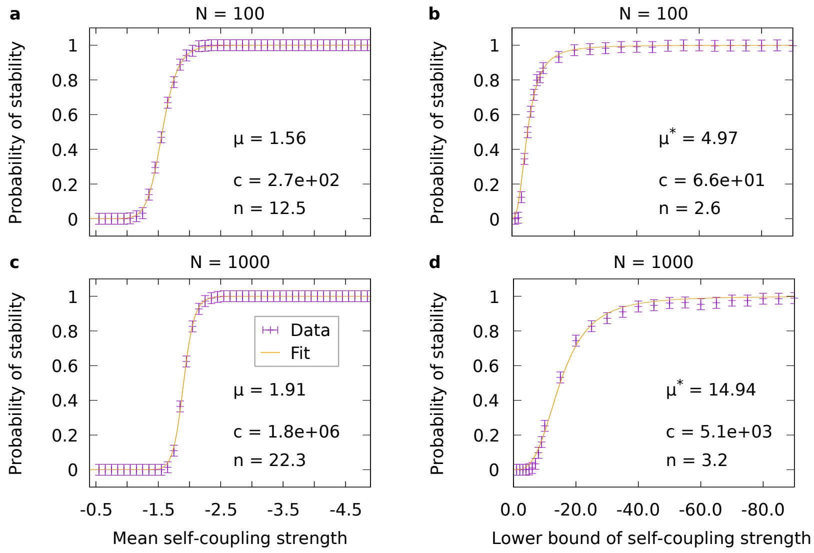

Supplementary Figure S5: **Examples of stabilization of networks generated with alternative preferential attachment parameters.** Networks of sizes $N = 100$ and $N = 1000$ are shown. Parameters for the preferential attachment algorithm (see Methods): $m = 4$ and $m_0 = 500$. **(a,c)** The stability over the mean self-coupling strength, where self-coupling strengths were drawn from intervals of varied position but fixed size 0.9. **(b,d)** The stability over the lower bound of the self-coupling strength, where the self-coupling strengths were drawn from an interval with the upper bound fixed at -0.4 and varying lower bound. The stabilization parameters μ and μ^* as well as the Hill fit parameters (cf. Eq. 1 in the main article) are given in the plots. For each self-coupling setting, the probability was averaged over 1000 trials. Error bars indicate the standard deviation estimated as described in Methods in the main article.

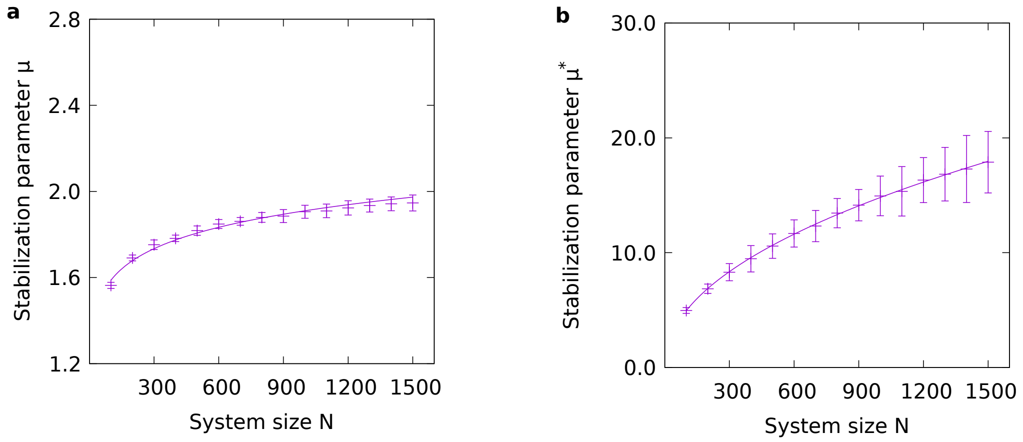

Supplementary Figure S6: **Stabilization parameter for networks with alternative preferential attachment parameters.** The stabilization parameter (computed from fits to data points obtained from 1000 trials, cf. Eq. 2 and Fig. S5) is plotted relative to the network size N. Parameters for the preferential attachment algorithm (see Methods): $m = 4$ and $m_0 = N/2$. The error bars show the error propagated from the fit parameters from which the stabilization parameter is computed. **(a)** Self-coupling strengths were drawn from an interval of fixed size and varying position. The stabilization parameter was obtained from the mean self-coupling strength. **(b)** Self-coupling strengths were drawn from an interval with fixed upper bound and varying lower bound. The stabilization parameter was obtained from the lower bound of the self-coupling strength. See Table 2 in the main article for the fit parameters and their uncertainty.

Characteristics of Kronecker-Leskovec networks

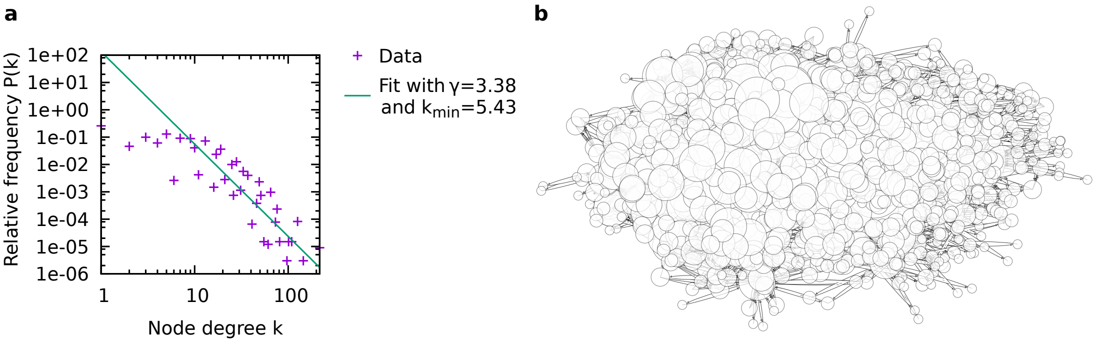

Supplementary Figure S7: **Generated Kronecker-Leskovec networks.** **(a)** Degree distribution of networks of size $N = 1000$, averaged over 1000 trials. The matrices were generated from seed matrices of dimension 10 that contained 82 zeros (see Methods). The green line shows a fit function as given by Eq. 10 in the main article. For fitting, all values with $k \geq 30$ were considered. The value of $\gamma = 3.38$ indicates similarity to scale-free characteristics (for which typically $2 \lesssim \gamma \lesssim 3$; Barabási, 2014). **(b)** Sample network of $N = 1000$ (Watts-Strogatz clustering coefficient: 0.000, average shortest path length: 3.80; also cf. Fig. 1a in the main article). Nodes are represented by circles and node degree is indicated by circle size. Arrows represent links between nodes. Self-coupling of nodes has been masked for better visibility.

References

Barabási, A.-L. 2014. The scale-free property, chapter 4. Freely available online under a CC BY-NC 3.0 license: http://networksciencebook.com/chapter/4.

Fienberg, S. E. 2012. A brief history of statistical models for network analysis and open challenges. *Journal of Computational and Graphical Statistics* 21:825–839.

Gardner, M. R. and Ashby, W. R. 1970. Connectance of large dynamic (cybernetic) systems: critical values for stability. *Nature* 228:784.

May, R. M. 1972. Will a large complex system be stable? *Nature* 238:413–414.
